# Burden of pelvic girdle pain during pregnancy among women attending ante-natal clinic, Ethiopia:a cross-sectional study

**DOI:** 10.1186/s12884-020-03184-4

**Published:** 2020-08-27

**Authors:** Moges Gashaw, Solomon Gedlu, Balamurugan Janakiraman

**Affiliations:** 1 Department of Physiotherapy, School of Medicine, College of Medicine and Health Sciences and Comprehensive Specialized Hospital, University of Gondar, P.O. Box: 196, Gondar, Ethiopia; 2 Department of Epidemiology and Biostatistics, Institute of Public Health, College of Medicine and Health Sciences, University of Gondar, Gondar, Ethiopia

**Keywords:** Pelvic girdle pain, Pregnancy, Antenatal care, Ethiopia

## Abstract

**Background:**

Pelvic girdle pain (PGP) is a commonly reported maternal morbidity that negatively impacts the well-being of women during pregnancy and extends long term into the post-partum period. The burden of maternal morbidity; including pregnancy-related PGP; has been overlooked in Ethiopia to date. This study aimed to determine the prevalence and identify factors associated with pelvic girdle pain during pregnancy in North West Ethiopia.

**Methods:**

A hospital-based cross-sectional study was conducted among pregnant women visiting the antenatal care clinic in Obstetrics ‘outpatient department at the University of Gondar comprehensive specialized hospital in Gondar. Data were collected by interview method using structured questionnaires, patient medical record reviews, and physical measurements. Univariate and multivariable logistic regression model analyses were used to identify factors associated with PGP.

**Results:**

A total of 424 participants with gestational ages ranging from 6 to 39 weeks participated in this study. The age of the study participants ranged from 18 to 44 years with a mean age of (27 ± 4.6 years). The overall cumulative prevalence of pelvic girdle pain among pregnant women was 103 (24.3%),95% CI (20.3, 28.8). The major associated factors with pelvic girdle pain were previous history of pelvic girdle pain (AOR 16.08; 95% CI, 8.47–30.51), previous history of back pain (AOR 1.66; 95% CI, 1.5–4.24) and having children (AOR 1.42; 95% CI, 1.29–3.76).

**Conclusion:**

One-quarter of pregnant Ethiopian women reported pelvic girdle pain. PGP must be considered as major pregnancy-related morbidity, and progress in the intervention of PGP is vital to enhance the quality of life in this population.

## Background

Maternal well-being has gained focus after global progress in maternal mortality reduction, and pelvic girdle pain (PGP) has garnered substantial interest by pelvic health professionals worldwide [1, 2]. According to the World Health Organization, maternal morbidity is defined as “any health condition attributed to and/or is aggravated by pregnancy and parturition that negatively impacts the women wellbeing” [3]. Maternal adaptation of musculoskeletal structures during pregnancy results in pregnancy-related musculoskeletal problems. However, most of them are not life-threatening conditions and are overlooked in LMICs with higher maternal mortality rate (MMR), disorders like pelvic girdle pain (PGP), low back pain (LBP), hip pain, and sacroiliac pain can impact women’s wellbeing. Even minor ailments can cause major concerns in this population [4, 5].

In Ethiopia, 80% of women reside in rural areas and women often experience physical hardship, such as carrying loads, agricultural labor, and domestic household work, in addition to raising children. On most occasions, Ethiopian women are involved in physical hardships during pregnancy. Like many other African countries, maternal morbidity and mortality are still unacceptably high, and maternal health is one of the top priorities in the Ethiopian national agenda. The global maternal mortality rate (MMR) 2015 is 216, the MMR of Sub-Saharan Africa (SSA) is 546, and Ethiopia is among those SSA countries with high MMR, 412. Research has shown an association between MMR and morbidity, where morbidity rates are much higher in countries with higher MMR. The government of Ethiopia (MOH, 2015) 2015–16 in the Health Sector Transformation Plan (HSTP) prioritized maternal health and well-being to promote women’s health in the country. The UNFPA report on maternal health in Africa estimated that for every woman who dies, 20–30 women suffer short-or-long term disabilities, including chronic pain. According to the EDHS 2016 report, only 17% of women attend post-natal care, which emphasizes the importance of estimating the burden of morbidity in this population during the ante-natal period [6–9].

Previous studies have shown that about 33–50% of pregnant women report PGP before 20 weeks of gestation and that the prevalence may reach 60–70% in late pregnancy [10–12].TheReported prevalence rates of pregnancy-related pelvic girdle pain (PPGP) in Western countries are 23.6, 22.6, 36.2, 14.2, 31.2, and 34% in various prospective studies, and 42.4 and 9.8% in retrospective studies [13–16]. The average reported prevalence of pregnancy-related pelvic girdle pain is 45.3% (range, 3.9–89.9%) in 28 studies [17, 18]. Studies have shown variation in the prevalence, severity, and factors concerning pregnancy, the attitude of women, socio-economic status, societal role, ethnicity, physical demand, cultural beliefs, and education status might influence how pregnancy-related PGP is perceived. Nevertheless, fear of movement, disability, negative psychological effects, history of LBP, age, parity, exercise levels, work history, job satisfaction, and education levels have been reported to be associated with PPGP in many countries [14, 16, 18–22]. There is an extensive reporting of maternal mortality and severe acute maternal morbidity in Ethiopia [23, 24], but the burden of chronic painful conditions including PPGP has not gained attention, our extensive search revealed scarce published regional works reporting the prevalence of morbidity-related conditions during pregnancy including PPGP in the sub-Saharan region, and none were found in Ethiopia.

Lack of awareness of the impairment caused by musculoskeletal disorders related to pregnancy can result in poor management of pregnant women. Most studies on pregnancy-related PGP have been conducted in developed countries [14, 16, 18], while these conditions are often ignored in developing countries. Hence, this study aimed to determine the prevalence and identify factors associated with PPGP among pregnant women in North West Ethiopia.

## Methods

### Study design, setting, and participants

A hospital-based cross-sectional study was conducted from January to June 2019 among pregnant women attending antenatal care clinic (ANC) in Obstetrics ‘outpatient department at the University of Gondar comprehensive specialized hospital (UoGCSH) in Gondar, Northwest Ethiopia, situated in the northern part of Ethiopia in Amharanational regional state, 747 km from the capital city of Addis Ababa at 12° 45’north latitude and 37 °.45’east longitudes with an elevation of 2706 m (8,878 ft) above sea level. Based on the 2016 population estimates of the Gondar City Administration Bureau, Gondar had total inhabitants of 335,000, among which 182,000 (52%) were women. The dominant means of livelihood is agriculture. About 77% of the rural mothers have to travel near to 20 km to get to public health services.

The Ethiopian public health system offers all women free health services during pregnancy. The University of Gondar Comprehensive Specialized Hospital provides tertiary care to the population of Gondar and its neighboring states, and most of the patients come from the lower socioeconomic strata. The Hospital has an antenatal clinic, which delivers antenatal care services every day. Annually, about 20,000 pregnant mothers visit the hospital for ANC services. The study population comprised of all pregnant mothers aged between 18 and 44 years, during any trimester of pregnancy those who were attending antenatal care during the data collection period at the ANC clinic, UoGCSH were considered. Pregnant women medically diagnosed with painful visceral pathologies, current low back pain, tuberculosis or syphilitic lesions of pubis, urinary tract infections, femoral vessel diseases, pre-eclampsia, eclampsia, serious intellectual disorders and recent trauma in the pelvic or low back region were excluded.

### Sample size determination and sampling procedure

A sample size of 442 was calculated using a single population proportion formula [25] based on the assumptions; 95% confidence interval, 50% prevalence (since there were no regional studies), 5% precision, 15% non-response, and contingencies. Systematic random sampling was used to select the participants based on the chart register of the day. On average, about 50–60 mothers visit the ANC care unit at UoGSCH per day. Based on the previous year ANC records the estimated number of visits by pregnant women during the study period would be 4040 and the derived K^th^ was 9. The first participant was selected between 1 and Kth by using the lottery method. The procedure was repeated until the estimated eligible sample size was attained.

### Study procedures

The study was conducted after obtaining ethical approval from the Institutional Review Board (IRB), College of Medicine and Health Sciences (CMHS), University of Gondar (Ref no; SOM/056/7/2019). Permissions were obtained from regional public health institutes and the authorities of the Department of Obstetrics and Gynaecology, UoGCSH, prior to the study. Before enrollment, the pregnant women were informed about the study, its objectives, and its importance. Written consent was obtained from the participants. A structured self-administered questionnaire (Additional file 1) was adopted from the pelvic girdle pain questionnaire [26] and other related literature [27–29]. To maintain consistency, the questionnaire was first translated from English to Amharic (the native language of the study area) and re-translated into English by professional translators and senior physiotherapists to check for the consistency of the questions and corrections were made accordingly. Four junior physiotherapist data collectors and two senior physiotherapist supervisors were selected. Before data collection, one-day intensive training on the objectives of the study, data collection procedures, and the confidentiality of information was given to data collectors and the supervisor by the primary investigator (MG). The questionnaire was also piloted on 5% (*n* = 22) of the total sample from the Maraki Health center, to evaluate the acceptability and applicability of the procedures and the questionnaire. Modifications and corrections of the measurement procedures were made based on analyses of pre-test data. The Cronbach’s alpha score was 0.82. Weight was measured using a floor checking weighing scale (Adam Equipment GFK 1320, Philips) with participants standing without shoes and wearing light clothing and recorded to the nearest 0.5 kg. Height was measured using a stadiometer while standing upright with the head in Seca 213 portable Stadiometer height and recorded with an approximation of 1 cm. The data collection process was closely monitored by the principal investigator (MG) and the supervisors throughout the data collection period.

### Definitions

PPGP was defined as ‘recurrent pain’ or continuous pain’ for at least a week between the posterior iliac crest and gluteal folds, particularly in the vicinity of the sacroiliac joints with/without pubic symphysis pain during the current pregnancy. A pregnant woman was considered to be suffering from PPGP if she responded “yes” to the specific question regarding localization of pain, which also included identifying the region on a flashcard shown during the interview.

Physical activity: Was categorized as ‘Yes’ or No based on the response to three questions: 1) Do you exercise regularly now? 2) If you exercise regularly, how many days a week do you exercise? 3) If you exercise regularly, how many minutes a day, on average, do you exercise? For question no, 1 the response alternatives were ‘yes’ or ‘no’ and for question no 2 the respondent has to fill the number of days/week and for question no 3 the respondent had to fill the average minutes of exercising per day. A respondent is defined to be physically active based on ACOG recommendations (1. ‘yes’, 2. ≥ 3 days, 3. ≥ 20 min).

Self-rated health: The pregnant women were asked to self-rate their health status now and before pregnancy. A ‘5’category responses were used to differentiate the responses arranged from very good through poor.

### Statistics

Data were entered using EpiInfo software version 7.1 and exported to the IBM Statistical Package for Social Sciences (SPSS) version 23 for the window for further analysis. Data entry with the original data was done by the data collector and the main investigator (MG) supervising each other to enhance correctness. Besides, the data was checked by two other researchers (BJ and SG) for completeness, accuracy, and clarity. Descriptive statistics, like frequencies and proportions, were computed, and the binary logistic regression model was used to identify significant variables. Variables with a *p-*value of less than 0.2 in the bivariable logistic regression analysis were entered into the multivariable logistic regression analysis. Both crude odds ratio (COR) and adjusted odds ratio (AOR) with the corresponding 95% confidence intervals were calculated to show the strength of the associations. Finally, a *p-*value of less than 0.05 in the multivariable logistic regression analysis was used to identify variables significantly associated with pelvic girdle pain. Potential confounding variables were entered into the model as covariates, and variability in the association was examined. When clear subgroups per category existed in the data, significance testing (Pearson χ^2^) and logistic regression model were performed for appropriately sized subgroups. Interaction terms were used to examine the potential association between independent variables and the dependent variable. Finally, the research was reported in adherence to the STROBE guidelines (Additional file 2).

## Results

### Sociodemographic characteristics of pregnant women

A total of 424 participants with gestational ages ranging from 6 to 39 weeks were included in this study. This is a 95.9% response rate and beyond the power calculated sample size. The reasons for non-responses were no time, not interested, and refusal husband to take consent. The age of the study participants ranged from 18 to 44 years with a mean age of (27 ± 4.6 years). Near above two-thirds of the pregnant women’s 306 (72.2%) were between the age group of 21 and 30 years. Most of them reported their religious affiliation as orthodox Christians (85.4%), 94.6% were legally married, and almost all of them (99.5%) reported no smoking habits. Only 4% of the participants reported to be unemployed, more than half (57.5%) the women had secondary level education and above, and very few (14.2%) practiced physical activity according to ACOG guidelines. The participant’s socio-demographic characteristics of pregnant women are presented in (Table 1).

**Table 1:** Socio-demographic characteristics of pregnant women who attained antenatal care clinic at Gondar, University Comprehensive Specialized Referral Hospital, Ethiopia (*n* = 424)

Variables	Categories	n	%
Age in years	< 20	36	8.5
(mean age (27 ± 4.6)	21–30	306	72.2
	> 30	82	19.3
Residence	Urban	364	85.8
	Rural	60	14.2
Marital status	Married	401	94.6
	Living in relationship	18	4.2
	Others ^a^	5	1.2
Religion	Orthodox Christian	362	85.4
	Muslims	45	10.6
	Others ^b^	17	4.0
Occupation	Homemaker	204	48.1
	Farmer	13	3.1
	Civil servant	98	23.1
	Merchant	51	12.0
	Unemployed	17	4.0
	Others^c^	41	9.7
Work status in week/hour	None	10	2.4
	0–20 h	172	40.6
	20–40 h	124	29.2
	more than 40 h	118	27.8
Work type	very heavy	8	1.9
	Heavy	52	2.3
	Fair	79	42.2
	Light	164	38.7
	Very light	21	5.0
Work satisfaction	very bad	6	1.4
	Bad	13	3.1
	Fair	60	14.2
	Good	271	63.9
	Very good	74	17.5
Level of education	No formal school	93	21.9
	Primary school	90	21.2
	Secondary school	82	19.3
	Diploma	87	20.5
	Degree and above	72	17.1
Income (ETB/month)	< 1000	122	28.8
	1000–2000	91	21.5
	2001–3000	82	19.3
	> 3000	129	30.4
Smoking habit	Never	422	99.5
	Past smoker	1	0.2
	Current smoker	1	0.2
Drinking alcohol habit	Never	309	72.9
	Past alcoholic	30	7.1
	Current alcoholic	85	20.0
Physical activity	No	329	77.8
	Yes	95	14.2
Self-rated health status	very good	219	51.7
	Quite good	122	28.8
	Fair	45	10.6
	Quite poor	28	6.6
	Poor	10	2.4

^a^ − divorced and singles; ^b^ − protestant, catholic;^c^-students and daily labours

### Obstetric-related characteristics

About half (47.4%) of the women were in their first pregnancy. Among the total study participant’s 94.1% of them had planned pregnancy, more than one third, 33.3% of them were in their second trimester (within the gestational weeks of 13 and 28) and more than half (52.6%) of the women had children. Nearly, one in three (29.02%) pregnant women had two or more gravidity; about 12.03% of the study participants had experienced abortion in their previous pregnancies and 11.3% reported taking one or more medications for different conditions. Among the total study participant’s 272 (64.2%) and 69 (16.3%) reported having a history of low back pain and pelvic girdle pain during their previous pregnancies, respectively. Table 2 shows the obstetric related characteristics of pregnant women.

**Table 2:** Obstetrics related characteristics of pregnant women who attained antenatal care services at Gondar, University Comprehensive Specialized Referral Hospital, Ethiopia (*n* = 424)

Variables	Categories	N	(%)
Trimesters	1st trimester(1-12wks)	3	0.7
(mean gestational week(31.52 ± 7.18)	2nd trimester(13-28wks)	141	33.3
	3rd trimester(29-40wks)	280	66.0
Number of previous Pregnancies	No	201	47.4
	One	100	23.58
	Two	95	22.4
	Three and above	28	6.62
Do you have children?	No	201	47.4
	Yes	223	52.6
History of abortion	No	373	89.97
	Yes	51	12.03
Pattern of current pregnancy	Planned	399	94.1
	Unplanned	25	5.90
Taking medication	No	376	88.7
	Yes	48	11.3
History of back pain	No	152	35.8
	Yes	272	64.2
History of PPGP	No	355	83.7
	Yes	69	16.3

*PPGP* Pregnancy related pelvic girdle pain, *wk* Weeks

### Pelvic girdle pain

One hundred and three (*n* = 103 (24.3%): 95% CI (20.3, 28.8) pregnant women reported experiencing pelvic girdle pain during the current pregnancy. The prevalence of pelvic girdle pain was significantly higher (61.2%, *n* = 63) among women who had children, in those who were in the aged range between 21 and30years (68.9%, *n* = 71). Among 103 pregnant mothers who reported to have endured PGP, (81.6%, *n* = 84) reported frequent pain. The burden of pelvic girdle pain was higher among urban dwellers 87(84.5%) and those women 85 (82.5%) who did not report to practice a recommended level of physical activity. Most of the women (67%, *n* = 69) who were in their third trimester reported PGP in the current pregnancy. The prevalence and distribution of pelvic girdle pain among pregnant women are shown in Table 3.

**Table 3:** Burden of pelvic girdle pain among pregnant women at Gondar, University Comprehensive Specialized Referral Hospital, Ethiopia (*n* = 424)

Variables	Categories	Pelvic girdle pain		χ^2^	P
		No (%)	Yes (%)		
Age in years	< 20	30 (9.3)	6 (5.8)	3.8	0.15
(mean age (27 ± 4.6)	21–30	235 (73.2)	71 (68.9)		
	> 30	56 (17.4)	26 (25.2)		
Residence	Urban	277 (86.3)	87 (84.5)	0.21	0.64
	Rural	44 (13.7)	16 (15.5)		
Marital status	Married	302 (94.1)	99 (96.1)	1.68	0.64
	Living in relationship	14 (4.4)	4 (3.9)		
	Others +	5 (1.5)	0 (0.0)		
Religion	Orthodox Christian	274 (85.4)	88 (85.4)	2.24	0.52
	Muslims	32 (10.0)	13 (12.6)		
	Others ++	15 (4.6)	2 (2.0)		
Occupation	Homemaker	156 (48.6)	48 (46.6)	3.9	0.58
	Farmer	9 (2.8)	4 (3.9)		
	Civil servant	73 (22.7)	25 (24.3)		
	Merchant	35 (10.9)	16 (15.5)		
	Unemployed	15 (4.7)	2 (1.9)		
	Others*	33 (10.3)	8 (7.8)		
Work status in week/hour	None	8 (2.5)	2 (1.9)	2.85	0.42
	0–20 h	137 (42.7)	35 (34.0)		
	20–40 h	89 (27.7)	35 (34.0)		
	> 40 h	87 (27.1)	31 (30.1)		
Work type	Very heavy	5 (1.6)	3 (2.9)	12.5	0.01
	Heavy	30 (9.3)	22 (21.4)		
	Fair	142 (44.2)	37 (35.9)		
	Light	126 (39.3)	38 (36.9)		
	Very light	18 (5.6)	3 (2.9)		
Work satisfaction	very bad	3 (0.9)	3 (2.9)	8.53	0.07
	Bad	7 (9.8)	6 (5.8)		
	Fair	41 (12.8)	19 (18.4)		
	Good	212 (66.0)	59 (57.3)		
	Very good	58 (18.1)	16 (15.5)		
Level of education	No formal school	69 (21.5)	24 (23.3)	3.64	0.45
	Primary school	72 (22.4)	18 (17.5)		
	Secondary school	59 (18.4)	23 (22.3)		
	Diploma	70 (21.8)	17 (16.5)		
	Degree and above	51 (15.9)	21 (20.4)		
Income (ETB/month)	< 1000	95 (29.6)	27 (26.2)	3.6	0.30
	1000–2000	71 (22.1)	20 (19.4)		
	2001–3000	65 (20.2)	17 (16.5)		
	> 3000	90 (28.0)	39 (37.9)		
Smoking habit	Never	321 (100)	101 (98)	6.26	0.04
	Past smoker	0 (0.0)	1 (1.0)		
	Current smoker	0 (0.0)	1 (1.0)		
Drinking alcohol habit	Never	242 (75.4)	67 (65.0)	4.8	0.08
	Past alcoholic	19 (5.9)	11 (10.7)		
	Current alcoholic	60 (18.7)	25 (24.3)		
Physical exercise	No	244 (76.0)	85 (82.5)	1.9	0.17
	Yes	77 (24.0)	18 (17.5)		
Self-rated health status	Very good	178 (55.5)	41 (39.8)	11.5	0.02
	Quite good	90 (28.0)	32 (31.1)		
	Fair	29 (9.0)	16 (15.5)		
	Quite poor	19 (5.9)	9 (8.7)		
	Poor	5 (1.6)	5 (4.9)		
Previous Pregnancies	No	161 (50.2)	40 (38.8)	8.4	0.03
	One	72 (22.4)	34 (33.0)		
	Two	73 (22.7)	21 (20.4)		
	Three and above	15 (4.7)	8 (7.8)		
Trimester	First	3 (0.9)	0 (0)	6.23	0.04
	Second	107 (33.4)	34 (33)		
	Third	210 (65.6)	69 (67)		
Do you have children	No	161 (50.2)	40 (38.8)	4.8	0.45
	Yes	160 (49.8)	63 (61.2)		
Taking medication	No	15 (88.2	33 (63.5)	3.7	0.07
	Yes	2 (11.8)	19 (36.5)		
History of back pain	No	124 (38.6)	28 (27.2)	4.5	0.04
	Yes	197 (61.4)	75 (72.8)		
History of PPGP	No	304 (94.7)	51 (49.5)	116.8	0.000
	Yes	17 (5.3)	52 (50.5)		
Frequency of PPGP (n 103)	Frequently	84 (81.6)			
	Occasionally	16 (15.5)			
	Rarely	03 (2.9)			

*ETB* Ethiopian birr, *PPGP* Pregnancy-related pelvic pain

### Regression analysis

In the univariate regression analyses, pelvic girdle pain was significantly (*p* < 0.20) associated with age, previous history of PPGP, previous history of back pain, current health status, physical exercise, and the number of children. Multivariate testing revealed that previous history of PPGP, previous history of back pain, and the number of children was significantly associated when adjusting for the other included variables. Table 4 shows the association between socio-demographic variables, obstetric and pregnancy-related variables, and pelvic girdle pain among the study participants. A previous history of PGP significantly increased the odds of PGP in current pregnancy by almost 16 times (AOR 16.08; 95% CI, 8.47–30.51). The model also showed that women reporting a history of back pain were more likely (AOR 1.66; 95% CI, 1.5–4.24) to have PGP. The adjusted odds of pelvic girdle pain were1.42 times higher among pregnant women who had children as compared to women who had no child (AOR 1.42; 95% CI, 1.29–3.76).

**Table 4:** Factors associated with pelvic girdle pain (PGP) pregnant women at Gondar, University Comprehensive Specialized Referral Hospital, Ethiopia (*n* = 424)

Variables	Categories	PGP		Univariate COR (95%CI)	Multivariate AOR (95%CI)
		No	Yes		
Age	≤ 20	30	6	2.3 (0.86–6.26)*	1.87 (0.54–6.53)
	21–30	235	71	1.54 (0.9–2.62)*	1.38 (0.69–2.77)
	> 30	56	26	1 ref	1 ref
Previous history of PPGP	No	304	51	1ref	1 ref
	Yes	17	52	18.2 (9.78–33.9)*	16.08 (8.47–30.5)**
Previous history of LBP	No	124	28	1ref	1ref
	Yes	197	75	1.68 (1.04–2.75)*	1.66 (1.5–4.24)**
Self-rated health status	Very good	178	41	4..34 (1.2–15.6)*	0.31 (0.02–4.6)
	Quite good	90	32	2.8 (0.76–10.36)*	1.24 (0.08–19.8)
	Fair	29	16	1.8 (0.45–7.2)	0.32 (0.02–5.72)
	Quite poor	19	9	2.1 (0.48–9.19)	1.84 (0.11–29.5)
	Poor	5	5	1ref	1ref
Physical activity	No	161	40	1.5 (0.84–2.63)*	1.6 (0.81–3.17)
	Yes	160	63	1ref	1ref
Having children	No	161	40	1 ref	1ref
	Yes	160	63	1.58 (1.1–2.49)*	1.42 (1.29–3.76)**

*variables significant with *p*-value < 0.2; ** variables significant with *p*-value ≤0.05, 1 = reference category; *COR* Crude odds ratio, *AOR* Adjusted odds ratio, *CI* Confidence interval, *PPGP* Pregnancy-related pelvic girdle pain, *LBP* Low back pain

## Discussion

The findings of this study showed that the overall prevalence of pelvic girdle pain among pregnant women was 24.3% with 95% CI (20.3–28.8). History of PGP, history of back pain, and women with one or more children were significant predictors of PGP. This could be due to the stress on the back and pelvic bones, an undue stretch, or cumulative damage of the pelvic soft tissues including the musculatures, and biomechanical changes that occur during the pregnancy [30].

The prevalence of pelvic girdle pain found in the current study is comparable with the results of the studies done in Australia (23%) [31], India (18.5%) [32], Iran (28.0%) [20], and another hospital-based cross-sectional study done India (29.9%) [29]. However, the reported prevalence of pregnancy-related PGP in our study was found to be lower (24.3%) compared to studies in developed countries (45–86%) [14, 17, 19, 33, 34] and the studies conducted in Indian tertiary care hospital (65%) [27] and Nepal (34%) [35]. The variation might be due to the difference in study settings, socio-economic status, level of education, and parity. Furthermore, the difference in maternal characteristics such as; most of the pregnant mothers in this study being in their third trimester (when the PPGP symptom seems to peak), with a mean gestation of 31 weeks, in contrast, most of the respondents in the reported studies who were in the second trimester, the gestational weeks of 16–40 weeks and there is vast variation in the outcome measures for e.g. some studies used self-report measures, such as pain location drawings and questionnaires, while others added physical examination to the self-reported measures to confirm the classification of PPGP like posterior pelvic pain provocation test, Patrick’s Faber test, palpation of the long dorsal sacroiliac joint ligament, Gaenslen’s test, distraction, compression, and Menell’s tests [16, 27, 35]. Palpation of symphysis and modified Trendelenburg’s test of the pelvic girdle were used to assess PGP and some of the studies were used modified oswestry disability index, pelvic girdle pain questionnaire, pain numbering scan and visual analogues scale to assess pain [16, 19, 27, 32].

However, the prevalence of this study is higher than the studies conducted in Netherland (7.3%) [16]. This difference observed in the prevalence rate of PGP could be due to the difference in study design, gestational week, and the study participant’s characteristics. The Netherland study was a longitudinal cohort study, reporting the prevalence, associated delivery-related and psychosocial factors, and consequences of self-reported pelvic girdle pain during and after pregnancy while the present study is a cross-sectional study.

The present study found that previous history of PGP and back pain has a significant association with the current pelvic girdle pain among pregnant women. The recurrence of PPGP with subsequent pregnancy, unknown risk factors, and lack of preventive measures are reported by several studies [16, 18, 36, 37]. The findings elsewhere also suggest that the relapse of PPGP is usually more prevalent, severe, and chronic, making PPGP a major public health problem and one of the significant maternal morbidity [38, 39]. Other studies, advocate the possible role of hormones like relaxin, progesterone on the pelvic girdle ligaments, and separation of the pubic symphysis [40]. However, there is a lack of consensus in the association of these factors with PPGP. Moreover, potential factors like altered muscle mechanics, strenuous work, history of low back pain, ethnicity, and the number of previous pregnancies are reported to be strongly associated with PPGP [38]. Predictably, having one or more children (multiple pregnancies) was found to be one of the significant predictors of PGP among pregnant women in this study and this finding is worrisome considering the higher (4.2 births per woman) fertility rate of Ethiopia [41]. This finding is consistent with the study done in Australia [42] and another cross-sectional study found that parity was significantly associated with PGP among pregnant women [20, 22]. The possible explanation might be, multiple pregnancies associated with altered musculoskeletal structures, degeneration, and repetitive stress of pelvic structures could result in chronicity and relapse of PGP. The absence of preventive measures, unknown aetiologies, and lack of specific interventions could also possibly result in high prevalence in this subgroup [18, 30].

Given that PPGP and possible reoccurrence of PGP among Ethiopian mothers is reasonably common, and the association of PGP with multiple pregnancy keeping in mind the higher fertility rate of Ethiopian women implicates the importance of further reporting of the burden of this chronic and disabling maternal morbidity in this population. For the benefit of future researches, there are some noteworthy limitations. First, pelvic girdle pain was self-reported with a recall period of gestational time without physical examination to the self-reported measures to confirm the classification of PPGP and some of the variables like psychological factors, postural assessment, and some of unmeasured societal or lifestyle variables were not considered. These confounders could lead to a possible variation in the estimation of association among PPGP and other variables. The cross-sectional nature of this study presents limitations in terms of causal association interpretations and long term effects through to the postpartum period. Future studies should address these concerns and determine causality and effect among pregnant women. Nevertheless, no prior study has directly examined the prevalence of PGP in pregnant women in Ethiopia and we strongly feel that these findings will provide more insight into the burden of PPGP in Ethiopia.

## Conclusion

About one-fourth of pregnant women in this study self-reported pelvic girdle pain at any point during pregnancy. Parity, previous pelvic girdle pain, and back pain are factors predicting the risk of PPGP. Reflecting on the facts like higher fertility rate, lack of utility of ANC, relapse and chronicity PGP, and absence of specific interventions PGP among Ethiopian pregnant women should be counted as a major women health problem. Pregnancy-related pelvic girdle pain is prevalent among Ethiopian pregnant women, and women who had a history of PGP were more likely to experience a relapse. PPGP significantly affects the role of being a mother, experience of pregnancy, family responsibility, societal role, and quality of life. Hence, this pregnancy-related condition must be considered as one of the major public health problems, especially in LMICs with high maternal mortality and severe acute maternal morbidity.

## Supplementary information

**Additional file 1.** English version questionnaire.

**Additional file 2.** STROBE statement checklist.

## Data Availability

All data relevant to our findings are contained within the manuscript. Requests for further details on the dataset and queries concerning data sharing shall be arranged based on a reasonable request to the corresponding author.

## Abbreviations

ACOG:ANC: Ante Natal Care
AOR: Adjusted Odds Ratio
BP: Back Pain
COR: Cruds Odds Ratio
EDHS: Ethiopian Demographic Survey
HSTP: Health Sector Transformation Plan
Km: Kilometer
LBP: Low Back Pain
LMICs: Low-middle income countries
MMR: Maternal Mortality Ratio
MoH: Ministry of Health
PGP: Pelvic Girdle Pain
PPGP: Pregnancy-related Pelvic Girdle Pain
SD: Standard Deviation
SSA: Sub-Saharan Africa
UNFPA: United Nations Population Fund
UoGCSH: University of Gondar Specialized Hospital
USA: United State of America

## References

[1] Elden H, Lundgren I, Robertson E (2013). Life’s pregnant pause of pain: pregnant women’s experiences of pelvic girdle pain related to daily life: a Swedish interview study. Sex Rep Healthcare.

[2] Campbell OM, Graham WJ (2006). Group LMSSs. Strategies for reducing maternal mortality: getting on with what works. Lancet.

[3] Firoz T, Chou D, von Dadelszen P, Agrawal P, Vanderkruik R, Tunçalp O (2013). Measuring maternal health: focus on maternal morbidity. Bull World Health Organ.

[4] Vermani E, Mittal R, Weeks A (2010). Pelvic girdle pain and low back pain in pregnancy: a review. Pain Practice.

[5] Foti T, Davids JR, Bagley A (2000). A biomechanical analysis of gait during pregnancy. JBJS..

[6] Abebe E, Singh K, Adefires M, Abraha M, Gebremichael H, Krishnan R (2014). History of low Back pain during previous pregnancy had an effect on development of low Back pain in current pregnancy attending antenatal care clinic of the University of Gondar Hospital, Northwest Ethiopia. J Med Sc Tech.

[7] Mohammed MA, Ahmed JH, Bushra AW, Aljadhey HS (2013). Medications use among pregnant women in Ethiopia: a cross sectional study. J of Appl Pharm Sci.

[8] Bekele A, Adefris M, Demeke S (2016). Urinary incontinence among pregnant women, following antenatal care at University of Gondar Hospital, north West Ethiopia. BMC Pregnancy Childbirth.

[9] Sahle G (2016). Ethiopic maternal care data mining: discovering the factors that affect postnatal care visit in Ethiopia. Health Inf Sci Syst.

[10] Gutke A, Östgaard HC, Öberg B (2006). Pelvic girdle pain and lumbar pain in pregnancy: a cohort study of the consequences in terms of health and functioning. Spine..

[11] Olsson C, Lena N-W (2004). Health-related quality of life and physical ability among pregnant women with and without back pain in late pregnancy. Acta Obstet Gynecol Scand.

[12] Robinson HS, Mengshoel AM, Bjelland EK, Vøllestad NK (2010). Pelvic girdle pain, clinical tests and disability in late pregnancy. Man Ther.

[13] Sneha Ashok D (2013). Pelvic girdle pain and low back pain during pregnancy and postpartum in indian women: a prospective cohort study of prevalence and risk factors in relation to pain intensity and disability.

[14] Kovacs FM, Garcia E, Royuela A, González L, Abraira V, Network SBPR (2012). Prevalence and factors associated with low back pain and pelvic girdle pain during pregnancy: a multicenter study conducted in the Spanish National Health Service. Spine..

[15] Verstraete E, Vanderstraeten G, Parewijck W (2013). Pelvic Girdle Pain during or after Pregnancy: a review of recent evidence and a clinical care path proposal. Facts Views Vision in ObGyn.

[16] Van De Pol G, Van Brummen HJ, Bruinse HW, Heintz APM, Van Der Vaart CH (2007). Pregnancy-related pelvic girdle pain in the Netherlands. Acta Obstet Gynecol Scand.

[17] Wu W-H, Meijer OG, Uegaki K, Mens J, Van Dieen J, Wuisman P (2004). Pregnancy-related pelvic girdle pain (PPP), I: terminology, clinical presentation, and prevalence. Eur Spine J.

[18] Robinson HS, Veierød MB, Mengshoel AM, Vøllestad NK (2010). Pelvic girdle pain-associations between risk factors in early pregnancy and disability or pain intensity in late pregnancy: a prospective cohort study. BMC Musculoskelet Disord.

[19] Mogren IM, Pohjanen AI (2005). Low back pain and pelvic pain during pregnancy: prevalence and risk factors. Spine..

[20] Mousavi SJ, Parnianpour M, Vleeming A (2007). Pregnancy related pelvic girdle pain and low back pain in an Iranian population. Spine..

[21] Bakker EC, van Nimwegen-Matzinger CW, Ekkel-van der Voorden W, Nijkamp MD, Völlink T (2013). Psychological determinants of pregnancy-related lumbopelvic pain: a prospective cohort study. Acta Obstet Gynecol Scand.

[22] Bjelland E, Eskild A, Johansen R, Eberhard-Gran M (2011). Pelvic girdle pain in pregnancy: the impact of parity. Obstet Anesth Dig.

[23] Berhan Y, Berhan A (2014). Causes of maternal mortality in Ethiopia: a significant decline in abortion related death. Ethiop J Health Sci.

[24] Woldemicael G, Tenkorang EY (2010). Women’s autonomy and maternal health-seeking behavior in Ethiopia. Matern Child Health J.

[25] Kasiulevičius V, Šapoka V, Filipavičiūtė R (2006). Sample size calculation in epidemiological studies. Gerontologija..

[26] Stuge B, Garratt A, Krogstad Jenssen H, Grotle M (2011). The pelvic girdle questionnaire: a condition-specific instrument for assessing activity limitations and symptoms in people with pelvic girdle pain. Phys Ther.

[27] Mahishale A, Borkar SS (2016). Determining the prevalence of patterns of pregnancy-induced pelvic girdle pain and low back pain in urban and rural populations: a cross-sectional study. J Sci Soc.

[28] Robinson HS, Mengshoel AM, Veierød MB, Vøllestad N (2010). Pelvic girdle pain: potential risk factors in pregnancy in relation to disability and pain intensity three months postpartum. Man Ther.

[29] Gupta M. Prevalence of pregnancy related pelvic girdle pain in Indian primigravida: a tertiary care hospital based study. Indian J Obstet Gynecol Res. 2014;1:16.

[30] Mahishale A, Borkar S (2015). Prevalence of patterns of pregnancy induced pelvic girdle pain and low Back pain in a tertiary care Centre-a cross sectional study. IJTRR..

[31] Pierce H, Homer CS, Dahlen HG, King J (2012). Pregnancy-related lumbopelvic pain: listening to Australian women. Nurs Res Pract.

[32] Bjelland EK, Eskild A, Johansen R, Eberhard-Gran M (2010). Pelvic girdle pain in pregnancy: the impact of parity. Am J Obstet Gynecol.

[33] Gutke A, Boissonnault J, Brook G, Stuge B (2018). The severity and impact of pelvic girdle pain and low-back pain in pregnancy: a multinational study. J Women's Health.

[34] Mens JM, Huis YH, Pool-Goudzwaard A (2012). Severity of signs and symptoms in lumbopelvic pain during pregnancy. Man Ther.

[35] Acharya RS, Tveter AT, Grotle M, Eberhard-Gran M, Stuge B (2019). Prevalence and severity of low back-and pelvic girdle pain in pregnant Nepalese women. BMC Pregnancy Childbirth.

[36] Aslan E, Fynes M (2007). Symphysial pelvic dysfunction. Curr Opin Obstet Gynecol.

[37] Vleeming A, Albert HB, Östgaard HC, Sturesson B, Stuge B (2008). European guidelines for the diagnosis and treatment of pelvic girdle pain. Eur Spine J.

[38] Elden H, Gutke A, Kjellby-Wendt G, Fagevik-Olsen M, Ostgaard H-C (2016). Predictors and consequences of long-term pregnancy-related pelvic girdle pain: a longitudinal follow-up study. BMC Musculoskelet Disord.

[39] Bergström C, Persson M, Nergård K-A, Mogren I (2017). Prevalence and predictors of persistent pelvic girdle pain 12 years postpartum. BMC Musculoskelet Disord.

[40] Aldabe D, Ribeiro DC, Milosavljevic S, Bussey MD (2012). Pregnancy-related pelvic girdle pain and its relationship with relaxin levels during pregnancy: a systematic review. Eur Spine J.

[41] Teshale AB, Tesema GA (2020). Magnitude and associated factors of unintended pregnancy in Ethiopia: a multilevel analysis using 2016 EDHS data. BMC Pregnancy Childbirth.

[42] Ceprnja D, Chipchase L, Gupta A (2017). Prevalence of pregnancy-related pelvic girdle pain and associated factors in Australia: a cross-sectional study protocol. BMJ Open.

