# Supplementary material for: Burden of pelvic girdle pain during pregnancy among women attending ante-natal clinic, Ethiopia:a cross-sectional study
Source: BMC Pregnancy Childbirth. 2020 Aug 27;20:494. doi: 10.1186/s12884-020-03184-4 (PMC7457245; doi:10.1186/s12884-020-03184-4)
Supplement: Supplementary file 1 — Additional file 1. English version questionnaire. [file 12884_2020_3184_MOESM1_ESM.docx]

# Appendix -I

## Annex I: subject consent form and English version questionnaire

Dears, Data collectors, please read the following statement to the respondents before you administer the interview/questionnaire

Dear Participants:

Good morning/afternoon? I am ……………………….. And I am one of the data collectors on behalf of the investigator

The physiotherapy department and Principal Investigator are jointly conducting this study in UOG. This study aimed at better understanding of the “the prevalence of PPGP and associated risk factors among pregnant women attending in ANC clinic of university of Gondar specialized hospital.Gondar,North West Ethiopia.

You have been chosen to participate in this study. The purpose of this questionnaire and assessment is to find out the information about self reported pelvic girdle pain among pregnant women attending in ANC clinic.

If you agree to participate in the study described above in the following question below

Yes ---------Continue administering interview

No -----------Give thanks to the participant and proceed to the next participant

***THANK YOU VERY MUCH FOR BEING PART OF THIS STUDY***

**Questionnaire [**English version Questionnaire],

Unique ID: _____________Date__________________

| **Survey information** | | | | |
| --- | --- | --- | --- | --- |
|  |  | | **Response** | **Cd** |
| **1** | Consent has been read and obtained | | 1. Yes 2. No | **I1** |
| **2** | Chart number | |  | **I2** |
| **3** | Interviewers ID | |  | **I3** |
| **4** | Date and time of data collection | |  | **I4** |
| **Socio-demographic questions** | | | | |
|  | **Question** | **Response** | | **Cd** |
| **101** | How old are you? (maternal age) | ................in years | | **S2** |
| **102** | Residence | 1.Urban 2.Rural | | **S3** |
| **103** | What is your highest level of education? | 1. No formal school 2. Primary school  3. Secondary school 4. Diploma  5. Degree& above | | **S4** |
| **104** | What is your religion? | 1. Orthodox 2. Protestants  3. Muslim 4. Others | | **S5** |
| **105** | What is your marital status? | 1. married  2.relation ship but not married  3.single  Others please specify | | **S7** |
| **106** | What is /was your occupation? | 1.House wife  2. Farmer  3. Civil servant  4.Merchant  5. Unemployed  6.other, please specify | | **S8** |
| **107** | Work status in weeks | 1.none 2.0–20 hours 3.20–40  hours 4. More than 40 hours | |  |
| **108** | Work type | 1. Very heavy 2. Heavy 3.fair 4.light 5.very light | |  |
| **109** | Work satisfaction | 1. Very bad 2. Bad 3. Fair 4,good 5.very good | |  |
| **110** | What is your monthly income? | .......................in Birr | | **S9** |
| **111** | Height | ………………in cm | | **S10** |
| **112** | Weight pre-pregnancy | ……………..kg | | **S11** |
| **113** | Weight after pregnancy | ……………..kg | |  |
| **114** | Gestational age in a week? | .........................in wk | | **S13** |
| **115** | Do you have children | 1. Yes 2. No | | **S** |
| **116** | If you yes how many children do you have | **………….** | |  |

| **Behavioral factor** | | | Response | | | | | Cd |
| --- | --- | --- | --- | --- | --- | --- | --- | --- |
| **201** | | How would you describe your smoking habit? | 1. Non smoker 2. Previous smoker 3. Current smoker | | | | | **B2** |
| **202** | | How would you describe your alcohol habit? | 1. Non alcoholic 2. Previously alcoholic 3. Currently alcoholic | | | | | **B3** |
| **203** | | Do you do physical exercise? | 1. Yes 2. No | | | | | **B4** |
| **204** | | If yes**B4** how many minutes/week? | in minutes | | | | | **B5** |
| **co-morbidity and general health** | | | | | | | | |
| **301** | Self rated health in the past 12 month | | | | 1.very good  2.quite good  3. fair  4.quite poor  5.poor | | **H1** | |
| **302** | Do you have previous history of back pain | | | | 1.yes  2.no | | H2 | |
| **303** | Do you have previous history of pelvic girdle pain | | | | 1.yes  2.no | | H3 | |
| **304** | Do you currently take medications? | | | | 1. Yes2. No | | **H4** | |
| **305** | If yes to M3, How many drugs do you take in a day? | | | | …….……in number | | **H5** | |
| **Pelvic girdle pain questionnaire** | | | | | | | | |
| **401** | Do you have any pain in b/n posterior iliac crest and gluteal fold? | | | 1. Yes 2. No | | **P1** | | |
| **402** | If yes question **F1**. Did you seek medical attention ? | | | 1. Yes 2. No | | **P2** | | |

|  | **How problematic is it for you because of your pelvic girdle pain to:** | **Not at all (0)** | **To a small extent (1)** | **To some extent (2)** | **To a large extent (3)** |  |
| --- | --- | --- | --- | --- | --- | --- |
| 1 | Dress yourself |  |  |  |  |  |
| 2 | Stand for less than 10 minutes |  |  |  |  |  |
| 3 | Stand for more than 60 minutes |  |  |  |  |  |
| 4 | Bend down |  |  |  |  |  |
| 5 | Sit for less than 10 minutes |  |  |  |  |  |
| 6 | Sit for more than 60 minutes |  |  |  |  |  |
| 7 | Walk for less than 10 minutes |  |  |  |  |  |
| 8 | Walk for more than 60 minutes |  |  |  |  |  |
| 9 | Climb stairs |  |  |  |  |  |
| 10 | Do housework |  |  |  |  |  |
| 11 | Carry light objects |  |  |  |  |  |
| 12 | Carry heavy objects |  |  |  |  |  |
| 13 | Get up/sit down |  |  |  |  |  |
| 14 | Push a shopping cart |  |  |  |  |  |
| 15 | Run |  |  |  |  |  |
| 16 | Carry out sporting activities* |  |  |  |  |  |
| 17 | Lie down |  |  |  |  |  |
| 18 | Roll over in bed |  |  |  |  |  |
| 19 | Have a normal sex life* |  |  |  |  |  |
| 20 | Push something with one foot |  |  |  |  |  |

*If not applicable, write “NA”

|  | **How much pain do you**  **experience:** | **None (0)** | **Some (1)** | **Moderate (2)** | **Considerable (3)** |  |
| --- | --- | --- | --- | --- | --- | --- |
| 21 | In the morning |  |  |  |  |  |
| 22 | In the evening |  |  |  |  |  |

|  | **To what extent because of**  **Pelvic girdle pain:** | **Not at all (0)** | **To a small extent (1)** | **To some extent (2)** | **To a large extent (3)** |  |
| --- | --- | --- | --- | --- | --- | --- |
| 23 | Has your leg/have your legs given way? |  |  |  |  |  |
| 24 | Do you do things more slowly? |  |  |  |  |  |
| 25 | Is your sleep interrupted? |  |  |  |  |  |
