# Supplementary material for: Burden of pelvic girdle pain during pregnancy among women attending ante-natal clinic, Ethiopia:a cross-sectional study
Source: BMC Pregnancy Childbirth. 2020 Aug 27;20:494. doi: 10.1186/s12884-020-03184-4 (PMC7457245; doi:10.1186/s12884-020-03184-4)
Supplement: Supplementary file 2 — Additional file 2. STROBE statement checklist. [file 12884_2020_3184_MOESM2_ESM.docx]

# STROBE checklist

# Title: Burden of pelvic girdle pain during pregnancy among women attending ante-natal clinic, Ethiopia: a cross-sectional study

**STROBE** Statement—checklist of items that should be included in reports of ***cross-sectional studies***

|  | Item No | Recommendation |
| --- | --- | --- |
| **Title and abstract** | 1 | 1. Study design is indicated in the abstract, methods section as an institutional-based cross-sectional study design. |
|  |  | (b) A balanced summary of what was done and what was found is provided in the methods and result section of the abstract |
| Introduction | | |
| Background/rationale | 2 | The scientific background and rationale for the investigation is reported. The rationale of the study was reported on 5^th^ paragraph of background section |
| Objectives | 3 | The general objectives of this study was stated as the final sentence of last paragraph under background session |
| Methods | | |
| Study design | 4 | It is presented in the first sentence of first paragraph of method section |
| Setting | 5 | A detailed description of the study area, location, and participant, described in the first and second paragraph of method section. |
| Participants | 6 | Eligibility criteria, the sources and methods of selection of participants are clearly stated in the last paragraph of methods session. |
| Variables | 7 | Both outcome and predictor variables are defined under operational definition sub-section in the methods in 1^st^ and 2^nd^para. |
| Data sources/ measurement | 8 | Source of data and data analysis methods are discussed in the 5^th^ and 6^th^ paragraphs of methods session |
| Bias | 9 | Efforts to address potential sources of bias were described in several part of method session. |
| Study size | 10 | Study size determination and sampling technique was mentioned under sample size determination sub-session in method section. |
| Quantitative variables | 11 | All quantitative variables treated as qualitative after categorizing them in one of most commonly used categories. |
| Statistical methods | 12 | (*a*) Statistical methods used in this study are described under data statistics sub-section in the last part of method session. |
|  |  | (*b*) Both sub group analysis and interaction terms were used. |
|  |  | (*c*) There were no missing data in this study |
|  |  | *(d)* Not applicable |
|  |  | (*e*) Not applicable |
| Results | | |
| Participants | 13 | (a) Number of participants, response rate is presented in the first paragraph of results session and detail socio-demographic characteristics in the table 1. |
|  |  | (b) About 95.9% participant responded and the most common reason for non-response is mentioned in the first paragraph of result section. |
|  |  | (c) This was cross-sectional study so; there is no flow as that of longitudinal study. |
| Descriptive data | 14 | (a) Characteristics of study participants (e.g. demographic, Obstetric related variables, ) and information on exposures and potential confounders is presented in tables 1 & 2. |
|  |  | (b) There were no missing data in this study. |
| Outcome data | 15 | Outcome variable (PPGP) described and summarized in table 3. |
| Main results | 16 | (*a*) Unadjusted estimates and confounder-adjusted estimates and their precision (eg, 95% confidence interval) are presented in table 4. Discussed under regression analysis 1^st^para in the result section. |
|  |  | 1. Category boundaries of continuous variables were categorized and reported in all tables. |
|  |  | (*c*) Regression model was used and expressed in odds ratio in table 4. |
| Other analyses | 17 | No clear or significant sub-group difference noted and interaction terms were used but non-significant. |
| Discussion | | |
| Key results | 18 | Key results to study objectives are discussed under discussion section with references. |
| Limitations | 19 | Limitations and possible strengths related to the current study are discussed in the final paragraph of discussion section on the way of viewing direction for researchers. |
| Interpretation | 20 | A cautious overall interpretation of results considering objectives, results from similar studies, and other relevant evidence is discussed under limitation of discussion section. |
| Generalisability | 21 | Generalisability (external validity) of the study results are mentioned under the conclusion section |
| Other information | | |
| Funding | 22 | Information regarding the source of funding (authors) and the role of the funders for the present study is presented under acknowledgment section. |
